# The Impact of Consumption Patterns on the Generation of Municipal Solid Waste in China: Evidences from Provincial Data

**DOI:** 10.3390/ijerph16101717

**Published:** 2019-05-16

**Authors:** Jinhui Liu, Qing Li, Wei Gu, Chen Wang

**Affiliations:** 1 School of Mechanical Engineering, University of Science and Technology Beijing, Beijing 100083, China; hndljh@163.com; 2 Donlinks School of Economics and Management, University of Science and Technology Beijing, Beijing 100083, China; qingl@xs.ustb.edu.cn (Q.L.); guwei@ustb.edu.cn (W.G.)

**Keywords:** municipal solid waste generation, consumption patterns, clustering analysis, provincial panel data

## Abstract

Municipal solid waste (MSW) is the derivative of urban development and it is harmful to the environment and residents’ health. But with sustainable MSW management, MSW can be applied as an important renewable energy. In order to achieve sustainable MSW management, it is necessary to understand the mechanism of MSW generation. Consumption patterns differ in various regions of China, which make the influencing factors of MSW have unique characteristics. To explore the factors influencing MSW generation in China, this study builds a global model based on the panel data of 30 Chinese provinces. Considering regional heterogeneity, provinces are clustered into three groups according to economic and consumption indicators. Each group has its own local model of MSW generation. The results show that household expenditure on housing and the tertiary industry proportion show opposite impacting directions in high-level and low-level provinces. Finally, with the combination of the grey model (1,1) (GM(1,1)) and multiple linear regression (MLR), we find that developing provinces will generate more MSW than developed regions. According to this, different provinces should control MSW by optimizing consumption pattern and efficient fiscal expenditure, and developing provinces should pay attention to MSW management and learn from the experience of developed provinces.

## 1. Introduction

Recently, urbanization, the development of the economy, and improved living standards and lifestyles of people have caused a sharp growth in municipal solid waste (MSW), especially in developing countries. Inappropriate or inefficient waste disposal methods will cause serious air, soil, and groundwater pollution, which will negatively affect the urban environment and threaten the health of residents [1]. Although MSW itself may have a negative influence on the economic development, recycling of MSW has the prospect of achieving economic and environmental benefits [2,3]. Moreover, with the development of improved technology, MSW can also be an important type of renewable energy, which can reduce the dependence on traditional fossil energy.

**Figure 1:**
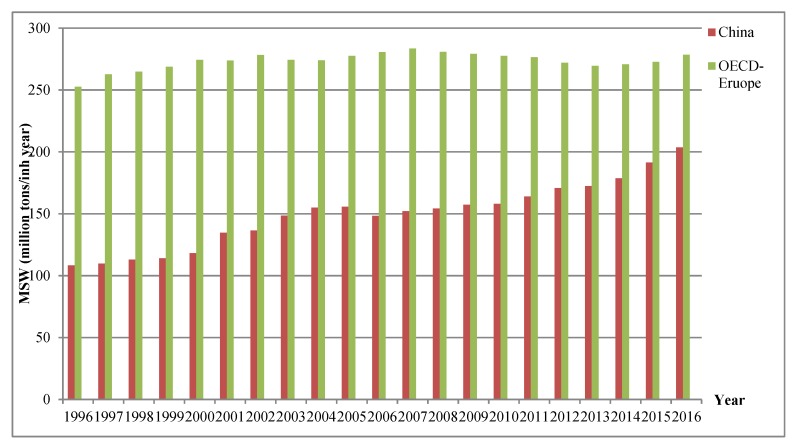
Comparison of municipal solid waste volume in China and Organization for Economic Co-operation and Development countries (Source: OECD statistics, https://stats.oecd.org/).

China is a producer of large volumes of MSW due to its large population and high population growth rate. According to the Organization for Economic Co-operation and Development (OECD) statistics, in 2016, the MSW generation volume in China reached 234 million tons, which is an increase of 88% compared to that in 1996, while the MSW volume in OECD European countries only increased by 10% during the period 1996–2016. As can be seen from Figure 1, the MSW volume in China shows an increasing trend but that in OECD European countries remains constant. By the end of 2016, China’s MSW volume had almost caught up with that of total MSW volume of all OECD European countries. Therefore, there is an urgent need for China to find an effective way to govern MSW and avoid the future consequences of rapidly accumulating MSW. To achieve urban sustainable development in China, recycling of MSW waste, harmless disposal technologies, and waste-to-energy technologies are all important issues to be addressed.

However, no matter which kind of MSW strategy is chosen, modeling MSW generation according to influencing factors is a very important problem in solid waste management, as it can provide more accurate predictions of future MSW generation [4]. In general, gross domestic product (GDP), population, and income are major factors. In recent years, some researchers have shown that consumption level is an important factor affecting the MSW volume [4,5]. However, some more unique and interesting factors may exist. In China, housing expenditure is an important factor affecting generation of MSW due to the large number of residents and limited housing areas. In regions with a relatively high level of economic development, such as in Beijing and Shanghai, housing expenditure accounts for more than 30% of residents’ total consumption expenditure, while in Sichuan or Anhui province, this proportion is less than 20%. The increase in housing expenditure will not only generate no MSW, but may reduce MSW volumes by reducing other spending. Variations in consumption behaviors of residents lead to variations in the overall expenditure pattern, which is an important factor affecting the MSW volume. By taking residents’ consumption behaviors into consideration, we can identify the generation mechanism of MSW. Moreover, based on socioeconomic factors, including expenditure patterns, the future trend of MSW generation in different Chinese regions can be more accurately depicted, so as to provide a theoretical reference for the relevant government departments to choose the most adaptive MSW management strategy.

It is crucial to have a clear understanding of MSW status before making MSW management plans. Waste treatment infrastructure usually requires a large amount of investment. Therefore, it is necessary to select the most economically viable and technically feasible MSW management strategy based on an accurate estimate of future MSW volumes. However, forecasting of waste generation is a challenging issue [6]. Some forecasting methods, like artificial neural networks (ANN), grey system, and time-series model are applied as reliable forecasting tools in the long-term forecasting of MSW generation [7]. But all these methods pay much attention to the time-series data of MSW and fail to take influencing factors from an area into account. In China, because of the inequality of economic and social development across regions, it is important to consider regional characteristics when forecasting the MSW generation in different areas.

To sum up, a clear understanding of the MSW generation mechanism and accurate prediction of MSW volumes are prerequisites in the development of strategies for MSW management. The generation mechanism of MSW varies according to differences in consumption patterns across regions. In exploring the impact of residents’ consumption behaviors on MSW volume, this paper uses panel data from 30 provinces in China to build regression models. Focusing on the relationship between regional influencing factors and MSW generation, three key questions will be answered in this paper:

1. Are there any particular factors influencing MSW generation in China?
2. How do these factors influence MSW generation in different regions of China?
3. What is the future volume of MSW based on the future trend of these influencing factors?

By answering the above three questions, our research enriches current literature on the influencing factors of MSW generation. In particular, the consideration of China’s unique consumption patterns may provide support for developing more reasonable waste management strategies. The paper is organized as follows: The second part is a review of relevant literature; the third part introduces data collection, description of variables, and model specification; the forth part analyzes the regression results of the country-wide model and three local models, and then forecasts MSW volumes of three typical provinces; the fifth part discusses the meaning and the possible reasons for the results; the last section concludes the paper.

## 2. Literature Review

MSW management is a popular research topic. Researchers have focused on the volume and composition of MSW, the influencing factors of MSW, and the prediction of MSW [8].

### 2.1. Factors Influencing MSW Generation

Many researchers have conducted studies into the relationship between MSW generation and socioeconomic/demographic factors, such as GDP [2], family or per capita disposable income [4,9,10], and consumption expenditure [4,5]. Wang and Geng [11] use the panel data of 31 provinces in China to reveal that the growth rate of tertiary industry, in terms of overall GDP and urban per capita disposable income, affect MSW carbon emissions indirectly and positively. Some studies conclude that with economic development and improvement in the standard of living, the increased consumption of food and clothing has caused growth in the volume of MSW generation [4,5,12]. In addition, Oribe-Garcia et al. [6] study municipalities grouped by means of similar socioeconomic characteristics to rebuild MSW generation models in different areas, and the results show that population density and the unemployment rate both reduce MSW generation. 

Studies often show that MSW volume is positively correlated with population density, consumption expenditure, and urbanization level [6,13]. The impact of income has not been consistent across studies in different countries. 

Some researchers use macroscopic income data to analyze its impact on MSW generation, such as Chen [9]. With an increase in personal disposable income, per capita volume of disposed municipal solid waste firstly declines, and grows at the second stage, and finally decreases again. They also find that income levels and social characteristics have an influence on the consumption patterns and MSW generation volumes and recycling volumes. Using sampling survey data, Qu et al. [14] divide household income into three categories to analyze the different household waste generation. They conclude that middle-income families generate more household waste than low-income and high-income families. Babayemi and Dauda [1] use random sampling to study different income levels and find that family income is positively related to per capita daily waste generation at the three socioeconomic levels. Gu et al. [10] use questionnaires to investigate the household income in Suzhou, China. The results show that the middle-income family has the highest waste generation rate. Grazhdani [15] studies the impact of income on waste generation based on a questionnaire survey in Prespa Park, suggesting that income has a positive association with waste generation rate and has a negative influence on recycling rate. Trang et al. [16] find that, because of the frequency of eating out, higher income households produce less household waste than lower income families. Ramachandra et al. [17] also acquire the data of household income by questionnaires, and they find that as the family income increases, the consumption rates increase, which in turn leads to more solid waste generation. 

There are other studies focusing on the relationship between some innovative socioeconomic factors and MSW generation. For example, Babayemi and Dauda [1] regard the social and economic status of citizens as having an effect on MSW generation. Prades et al. [18] use the independent variable of people:car ratio to represent the purchasing power of residents. They find that the people:car ratio has a negative effect on MSW generation. 

The influencing factors, analysis methods and models, and related references are listed in Table 1. It can be seen from the previous studies that MSW generation mainly depends on the level of urbanization and specific socio-economic characteristics [11,13]. In the literature, more attention is paid to the impact of residents’ income on MSW production instead of consumption behavior. This cannot reveal the influence of different consumption patterns on MSW generation. It is proposed that residents’ consumption level and consumption patterns will not only affect the MSW volumes, but also affect the composition of MSW [19]. Therefore, to accurately depict the features of MSW generation and develop appropriate MSW management strategies, it is important to consider the impact mechanism of residents’ consumption behavior on MSW generation. 

### 2.2. Forecasting Methods for MSW

MSW management is a complex system [26], and the precise forecasting is a foundation for successful management [27]. Therefore, the consideration of total volumes and patterns of MSW generation is indispensable [28]. As listed in Table 2, methods of regression, time-series analysis, grey models, scenario analysis, and artificial neural network (ANN) are widely used methods in research on MSW forecasting [26].

Regression models are widely used to forecast MSW generation. Factors, such as GDP [7,19], population [29], and urbanization [30] are used in regression models. This method of forecasting shows relatively low accuracy because it cannot encompass all the factors influencing MSW generation. Some literatures try to achieve better results by considering the seasonality in MSW generation, and they combine seasonal autoregressive moving average (SARIMA) with time-series analysis. But this method requires large scale time-series data [29,31]. Scenario analysis sets baseline scenarios and different possible scenarios to add qualitative analysis to quantitative research [32]. 

Recently, grey models (GMs) and artificial neural networks (ANNs) have been used in the forecasting. Although the grey system does not take influencing factors into consideration, the GM can provide a reasonably accurate result even with a lack of long-term data [32]. GM(1,1) showed better accuracy and easier application in forecasting MSW [30]. The combination of GM and SARIMA models shows a better result in terms of month-scale and long-term forecasting of MSW generation [30]. Without considering any influencing factors, ANN are applied as a reliable tool for the forecasting of MSW volumes using different time scales. Abbasi and El Hanandeh [27] use four intelligent system algorithms, namely support vector machine (SVM), adaptive neuro-fuzzy inference system (ANFIS), ANN, and k-nearest neighbors (kNNs), to predict monthly waste generation. The result shows that ANN models could be appropriately used to establish MSW generation forecasting models. 

In this paper, we will pay close attention to the relationship between MSW generation and consumption patterns. It is anticipated that the generation characteristics of MSW can be better forecast using the consumption patterns of Chinese residents rather than income. Subsequently, in this paper, the GM is combined with the multiple linear regression model (MLR) to forecast future MSW generation. By doing so, we can not only obtain more accurate forecasting data of influencing factors of MSW generation using GM(1,1), but we can take all these factors into account to forecast the future MSW volume using MLR.

### 2.3. Contributions of This Paper

Following in the footsteps of previous studies, this paper aims to make contributions in three ways: (1) Instead of considering the total amount of income and consumption expenditure, we focus on the impact of consumption patterns on MSW generation. China’s consumption patterns have unique characteristics, such as the high proportion of expenditure on housing in economically-developed regions. Such unique consumption patterns may have interesting impacts on MSW, which have not been studied in previous literature. (2) Instead of using the typical Chinese east, middle, and west division, we divided 30 provinces in China into three clustering groups, according to economic and consumption indicators. Based on this division method, the analysis results provide greater insight into the generation mechanism of MSW. (3) We combine the GM(1,1) model with the MLR model not only to improve the forecasting ability but also to display relationships between MSW generation and influencing factors.

## 3. Materials and Methods

### 3.1. Data

The study areas selected are Chinese provincial administrative regions, and the data used in this paper was derived from 2008–2017 China Statistical Yearbooks. In this study, provincial panel data was used to analyze the relationship between MSW generation and socioeconomic factors. Because of the missing data for Tibet, it was excluded from our study. The other 30 Chinese mainland provinces were selected for this study. The economic and consumption data used 2007 as the baseline to eliminate errors in price fluctuations in different years. In addition, to reveal the correlation coefficient between different socioeconomic factors and MSW generation, we adjusted the measuring units of the relevant data. All the variables involved in this paper are described in the following section.

#### 3.1.1. Dependent Variable: Municipal Solid Waste (MSW)

As accurate MSW generation volumes cannot be measured directly in developing countries [32], it was roughly estimated using the clearance volume of MSW for the 30 provinces of China. Although the amount of MSW clearance collection was less than the true amount of MSW generation, it could be used to reflect the trend in MSW generation.

#### 3.1.2. Core Independent Variables

1. Financial expenditure, reflected by the variable of “expenditure for general public services” (PSE). Vieira and Matheus [34] suggest that the inequality of society and economy among regions may affect MSW generation. In this study, PSE was selected to represent the regional heterogeneity of financial expenditure, which can reflect the inequality in social development across different regions.

2. Consumption patterns, reflected by the variables of average annual per capita consumption expenditure of urban households on food, clothing, and housing. The consumption patterns and food expenditure are important influencing factors in MSW generation [17,35]. Variations in consumption patterns and lifestyle lead to variations in the generation and composition of MSW [5]. However, there are few studies that consider the consumption pattern as one of the influencing factors in MSW generation. Therefore, in this paper, consumption patterns were reflected by three variables: The average annual per capita consumption expenditure of urban households on food (FC), clothing (CC), and housing (HC). Housing expenditure was considered as a factor unique to China in terms of MSW generation and was introduced innovatively in this study to explore its impact on MSW generation.

#### 3.1.3. Control Variables

1. Per capita GDP (PGDP). GDP or PGDP has been frequently used in the research into MSW generation [2,19]. In this paper, PGDP was used to reflect the economic status. The real GDP was calculated to be the constant price in 2007.

2. Population density (PD). This is another typical factor considered in previous studies [6,23], and is calculated by dividing urban population by urban built-up area.

3. The tertiary industry proportion (TIP) is calculated as the proportion of added-value of the tertiary industry in terms of the general GDP. Some researchers suggest the TIP is closely correlated with the generation of municipal solid waste [11], as the tertiary industry provides household commodities. 

4. Age structure (AGE). The age structure has long been considered in municipal solid waste generation [7,8]. Here, we used the population dependency ratio to represent the age structure of each province. The population dependency ratin includes the child dependency ratio and the dependency ratio of the elderly. This indicator not only unifies the data dimension but also reflects the family structure.

5. Family size (FS). Family size is correlated to waste generation because of its influence on eating and consuming habits [5,16,36]. Here, family size was measured by the average population of each household.

Table 3 shows the statistical description and units for all variables used in this study.

### 3.2. Research Framework and Models

**Figure 2:**
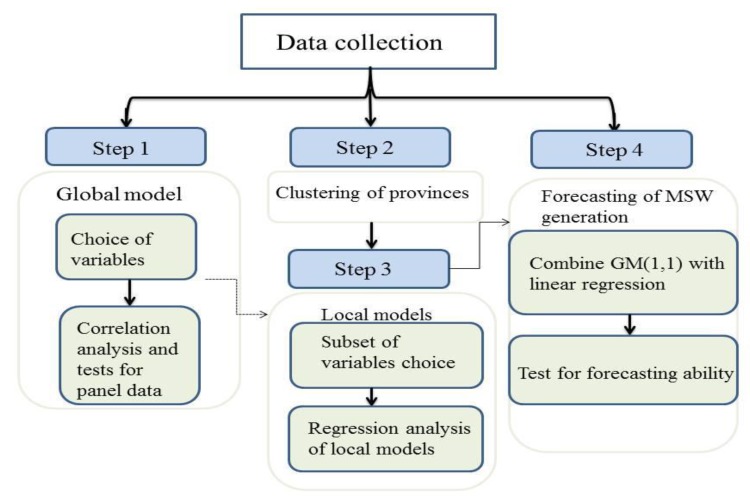
The flowchart for the methodology used in model building and forecasting of MSW generation.

After collecting data, we first chose variables to establish a global model for MSW generation in 30 Chinese mainland provinces (Step 1). Subsequently, to reveal the regional heterogeneity in different provinces, we clustered provinces into three different groups according to economic and consumer indicators (Step 2). Aiming to identify economic and consumption factors regarding MSW generation for each group of provinces, the local models were rebuilt based on re-selected factors (Step 3). Finally, the local models were used to forecast the MSW generation in five years in the corresponding areas. The forecasting ability was also assessed (Step 4). These steps can be seen in Figure 2.

#### 3.2.1. Step 1: Regression Analysis of Global Model

According to what has been mentioned before, a global model (Equation (1)) was established, which includes variables of PSE, FC, CC, HC, PGDP, PD, and TIP.

Global Model:

(1) $$\begin{array}{l} {\mathrm{MSW}}_{it}=\beta_{0}+\beta_{1}PSE_{it}+\beta_{2}FC_{it}+\beta_{3}CC_{it}+\beta_{4}HC_{it}+\beta_{5}PGDP_{it}+\beta_{6}PD_{it}+\beta_{7}TIP_{it}+\beta_{8}AGE_{it}\\ \;+\beta_{9}FS_{it}+C_{i}+Y_{t}+u_{it} \end{array}$$

In the global model, $\beta_{0}$ denotes constant; $\beta_{1}–\beta_{9}$ are, respectively, the coefficients of PSE, FC, CC, HC, PGDP, PD, TIP, AGE, and FS; $C_{i}$ represents individual effects in province *i*; $Y_{t}$ represents a time effect; and $u_{it}$ is an error term, *i* denotes province *i,* and *t* denotes year *t*. For example, ${\mathrm{MSW}}_{it}$ denotes the amount of municipal solid waste generation of province *i* in year *t*. The amount of MSW clearance volume is used to represent the amount of MSW. 

Variance inflation factor (VIF) test and correlation analysis are applied to check multicollinearity and correlation strength among variables. As shown in Table A1 and Table 4, because all VIFs are less than 10 and the mean VIF is less than 5, we can conclude that the multicollinearity would not seriously affect our regression results [37].

To ensure the accuracy of regression results, least square dummy variable model (LSDV), the Hausman test, the heteroscedasticity test, serial correlations, and the cross-sectional dependence test can help to choose the most appropriate method from mixed OLS, fixed effects, and random effects. As shown in Table 5, the result of the Hausman test shows *p* value is 0, so we can conclude that the fixed effects model is a better fit than the random effects model according to Hausman et al. [38]. Considering the problem of serial correlation, cross-sectional dependence, and heteroscedasticity in our panel data, we used the robust fixed effect model to solve the possible problems that may exist in the data.

#### 3.2.2. Step 2: Clustering of Provinces: Regional Heterogeneity of MSW Generation

Many studies have shown that there are regional heterogeneities among regions with different economic development levels [39,40]. Especially, MSW generation differs among regions with different socioeconomic levels. So, it is necessary to build corresponding models for MSW generation of regions with similar socioeconomic development levels [6].

In Step 2, we used the average value of per capita GDP and per capita consumption expenditure of urban residents in 2007–2016 to cluster the 30 provinces. By taking the study of Ahmad and Dey [41] as a reference, the k-mean method was used to cluster the 30 provinces into three groups, namely high, middle, and low economic and consumption level. The reason for re-clustering the provinces instead of using the typical division is because the typical one is more about geographical features not socioeconomic differences. This clustering algorithm applied an iterative method to group the data into a pre-determined *k* (here *k* = 3) number of clusters by minimizing a cost function θ of the type (Equation (2)).

(2) $$\theta =\overset{n}{\underset{i=1}{\sum }}{\left\|d_{i}-C_{j}\right\|}^{q}$$

where $C_{j}$ is the center of jth cluster and is the center nearest to data target $d_{i}$; n is the number of elements in data; q is an integer defining the properties of distance function. For numeric data sets, the cluster center can be represented by the mean value of every characteristic [41].

#### 3.2.3. Step 3: Regression Analysis of Local Models

Many socioeconomic factors may affect MSW generation. However, because of heterogeneity among provinces at different economic and consumption levels, a MSW generation model should be built for each respective clustering group. In Addition, for each group, corresponding variables should be re-selected from the eight variables above. After several attempts, the following three local models were developed in Equations (3)–(5) with better performance:

Local Model 1:

(3) $${\mathrm{MSW}}_{H}=\beta_{0}+\beta_{1}PSE_{it}+\beta_{2}FC_{it}+\beta_{3}HC_{it}+\beta_{4}PGDP_{it}+\beta_{5}TIP_{it}+\beta_{6}FS_{it}+C_{i}+Y_{t}+u_{it}$$

Local Model 2:

(4) $$\begin{array}{l} {\mathrm{MSW}}_{M}=\beta_{0}+\beta_{1}PSE_{it}+\beta_{2}FC_{it}+\beta_{3}CC_{it}+\beta_{4}HC_{it}+\beta_{5}PD_{it}+\beta_{6}TIP_{it}+\beta_{7}AGE_{it}+C_{i}+Y_{t}\\ \;+u_{it} \end{array}$$

Local Model 3:

(5) $${\mathrm{MSW}}_{L}=\beta_{0}+\beta_{1}PSE_{it}+\beta_{2}FC_{it}+\beta_{3}HC_{it}+\beta_{4}PD+\beta_{5}TIP_{it}+C_{i}+Y_{t}+u_{it}$$

As in Step 1, the fixed effects mode is chosen here according to the results of the multicollinearity test (Table A2) and other necessary tests (Table 5). Driscoll–Kraay standard errors [42] and the robust rixed model were applied in local models.

#### 3.2.4. Step 4: Forecasting of MSW Generation

The grey model GM(1,1) and MLR model are the two mainstream methods for forecasting and both have advantages and disadvantages. GM(1,1) can provide a reasonable result of time-series without revealing the relationship between the MSW generation and certain socioeconomic factors. MLR models can consider important influencing factors but not all the factors, leading to reduced accuracy of forecasting. To solve this problem, Xu et al. [30] propose a hybrid model that combines the SARIMA model with grey forecasting models to forecast MSW generation at various time scales. This paper applied this thought to combine the GM(1,1) with the MLR model to forecast the future trend of MSW generation. GM(1,1) was used to forecast the trend of influencing factors, such as FC and CC, per capita GDP, etc. Each factor could be seen as an independent time-series. The future values of influencing factors were then used as variables in MLR. Using this method, a better forecasting result could be achieved while revealing the relationship between MSW generation and its influencing factors.

The forecasting ability of forecasting models was evaluated by the mean absolute percentage error (MAPE) and R^2^, which were computed as Equations (6) and (7). $y_{i}$ is the observed true value, $f_{i}$ is the forecasted value for province *i*, and *n* is the number of the observations [6]. MAPE can use the acquired data to reflect the degree of deviation from the predicted value to the observed true value. The smaller the MAPE is, the better the forecasting ability will be.

(6) $$\mathrm{MAPE}=\frac{1}{n}\overset{n}{\underset{i=1}{\sum }}\left|\frac{y_{i}-f_{i}}{y_{i}}\right|$$

R^2^ is the coefficient of determination, representing to what extent the variation in the dependent variable can be explained by the variation in the independent variable. The closer R^2^ is to 1, the better the degree of fit of the regression equation is.

(7) $$R^{2}=1-\frac{\sum_{i=1}^{n}(y_{i}-{\hat{y)}}^{2}}{\sum_{i=1}^{n}(y_{i}-{\bar{y)}}^{2}}$$

In this paper, using the MSW generation data during 2007–2016, the GM(1,1) and MLR methods were combined together to forecast the MSW generation of three province groups in five years. MAPE and R^2^ were used to test the forecasting ability. 

## 4. Results

### 4.1. Regression Results of Global Model

The robust fixed effects model was used in the global model. The global model for MSW generation was developed as follows:

(8) $$\begin{array}{l} \mathrm{MSW}=519.923+0.738\;\mathrm{PSE}+0.194\;\mathrm{FC}-0.564\;\mathrm{CC}-0.62\;\mathrm{HC}+0.008\;\mathrm{PGDP}-0.027\;\mathrm{PD}\\ \;-2.254\;\mathrm{TIP}-3.066\;\mathrm{AGE}+123.279\;\mathrm{FS} \end{array}$$

Table 6 shows regression results of the global model. With the independent variables PSE, FC, CC, HC, PGDP, PD, TIP, AGE, and FS, the model was able to explain 53.8% of the reasons for MSW generation. Except for TIP, all these nine independent variables had close relationships in the model, with every coefficient being significant. 

### 4.2. Results of Clustering

**Figure 3:**
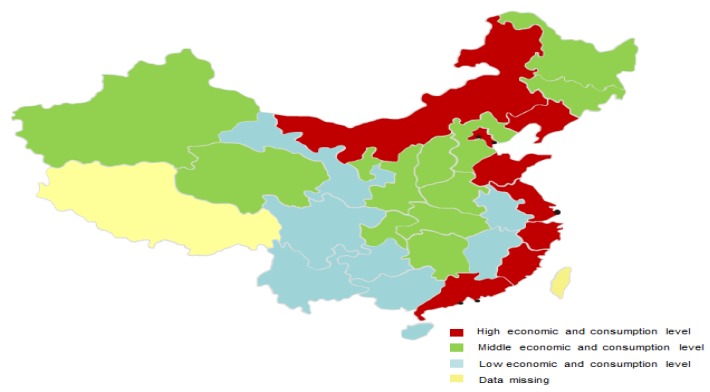
Clustering results of 30 provinces according to economic and consumption indicators.

Here, we applied the k-mean clustering method to divide the data set of 30 provinces into three groups according to the indicators of per capita GDP and per capita consumption expenditure. The results of clustering can be seen in Figure 3. 

1. Cluster 1: W total of 10 provinces with high economic and consumption levels, including Beijing, Tianjin, Inner Mongolia, Liaoning, Shanghai, Jiangsu, Zhejiang, Fujian, Shandong, and Guangdong provinces.

2. Cluster 2: W total of 12 provinces with middle economic and consumption levels, including Jilin, Chongqing, Heilongjiang, Hebei, Hubei, Shaanxi, Henan, Shanxi, Hunan, Qinghai, Sinkiang, and Ningxia provinces. 

3. Cluster 3: A total of eight provinces with low economic and consumption levels, including Sichuan, Anhui, Hainan, Gansu, Guangxi, Yunnan, Kweichow, and Jiangxi provinces.

### 4.3. Regression Results of Local Models

The local models of MSW generation for the three groups are shown as Equations (9)–(11). Table 6 shows the main statistics in local models. These three local models could explain 70.4%, 34.43%, and 76.42% for the high, middle, and low groups, respectively. In general, the local models had better explanatory ability than the global model.

However, the explanatory power of the MSW generation in the middle economic and consumption cluster was poorer than in the other two local models. The same problem can also be observed in regional MSW generation models in other research [6]. It is believed that this poor explanatory power may be due to the exclusion of some unique explanatory variables behind the waste generation in different regions. According to this, we consider that some of the selected socioeconomic factors cannot fully explain the MSW of the middle cluster. In this paper, some influencing factors, such as geographical location characteristics and living habits, were not included in the local models.

(9) $${\mathrm{MSW}}_{H}=586.004+1.421\;\mathrm{PSE}+0.444\;\mathrm{FC}-2.364\;\mathrm{HC}+0.01\;\mathrm{PGDP}-7.353\;\mathrm{TIP}+212.72\;\mathrm{FS}$$

(10) $$\begin{array}{l} {\mathrm{MSW}}_{M}=653.157+0.473\;\mathrm{PSE}+0.526\;\mathrm{FC}-2.393\;\mathrm{CC}+1.131\;\mathrm{HC}-0.051\;\mathrm{PD}+0.32\;\mathrm{TIP}\\ \;-5.526\;\mathrm{AGE} \end{array}$$

(11) $${\mathrm{MSW}}_{L}\;=-161.416+0.223\;\mathrm{PSE}+0.604\;\mathrm{FC}-2.299\;\mathrm{HC}+0.013\;\mathrm{PD}+2.193\;\mathrm{TIP}$$

### 4.4. Forecasting Ability of MSW Generation

We evaluated the forecasting ability of MLR models before forecasting the future trend of MSW generation. The real data of influencing factors in 2007–2016 is used in the local models to forecast the MSW generation in the three clusters. Zhejiang, Hubei, and Sichuan are chosen from cluster 1, 2, and 3, respectively, for the test of forecasting ability, because these provinces have the same MSW volumes in 2016 (about 900 ten thousand tons), and, as such, the forecasting volume of MSW generation will reflect the impact of the changes of different socioeconomic characteristics. The detailed results on forecasting ability can be seen in Table 7.

In local model 1, the MAPE and R^2^ of Liaoning province are 10.98% and 0.88; in local model 2, the MAPE and R^2^ of Hubei province are 10.68% and 0.93; and in local model 3, the MAPE and R^2^ of Sichuan are 5.20% and 0.99. Therefore, we can safely conclude that these three local models have ideal forecasting ability by including influencing factors into models.

### 4.5. Forecasting of MSW Generation in Five Years of Three Different Clusters

**Figure 4:**
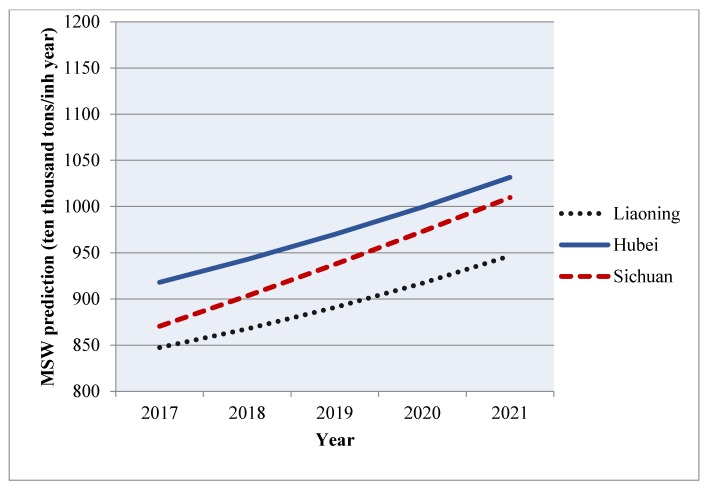
Forecasting results of MSW generation in three provinces during 2017–2021.

After proving the forecasting ability of the three local models, we used them to forecast the MSW generation in three clustering provinces in five years. As is shown in Figure 4, MSW generation in these three provinces showed an overall growth trend. MSW generation in Liaoning will reach 946.59 ten thousand tons, with the smallest growth rate; in Sichuan, it will reach 1009.82 ten thousand tons; and in Hubei, it will reach 1031.61 ten thousand tons in 2021. Although Hubei province will generate the largest MSW volume in 2021, Sichuan province has the fastest growth rate of 20.24% during 2016–2021.

## 5. Analysis and Discussion

### 5.1. MSW Generation in the Global Model

The regression results of the global model tell us much about the relationship between MSW generation and some important influencing factors. To begin with, PSE had a significant and negative coefficient in the model. It means that the more the government invests in general public services, the more the MSW will be produced. This may be because we use the amount of solid waste clearance to represent the MSW generation. The expenditure on general public services can help municipalities enhance the collecting efficiency. Expenditure on general public services can support all relevant governments to perform their functions, and; thus, has huge impact on MSW clearance and collection. 

The same as Bosire et al. [4] and Han et al. [5], food consumption was proved to correlate positively with MSW generation. However, the consumption expenditure on clothing and housing correlated negatively with MSW generation. Clothing goods are often thrown away after being used for a relatively longer time. Meanwhile, because of the existence of clothes recycling bins in residential communities, the old clothes may not be collected as waste. With a significant and negative coefficient, the housing expenditure showed even more interesting results. As mentioned by Lau and Li [43], housing expenditure can pose a heavy load on family and affect the real disposable income of urban residents. Therefore, it makes residents consume less food and generate less waste. 

As for the control variables, per capita GDP and PD also showed good results with significant coefficient. GDP has always been seen as an important factor which has a positive impact on MSW generation [2,11]. PD was proved to have negative impact on MSW generation, which is the same as the results of Oribe-Garcia et al. [6]. This may result from the scale effect, which means that the larger household size may produce less per capita MSW volumes. Without considering other influencing factors, provinces with higher population density are likely to produce less MSW. Provinces with higher population densities should use this advantage to better improve their MSW management. Garbage classification and recycling systems should be implemented, which can not only reduce actual waste generation but also promote efficient re-use of resources. Provinces with low population density tend to produce more MSW due to the lack of the scale effect in MSW generation. Such provinces can reduce unnecessary MSW production by advocating re-using awareness to residents. Especially for provinces with high population density and imperfect waste disposal infrastructure, the garbage classification and recycling systems can effectively promote waste recycling and waste-to-energy. For example, establishing the four-category garbage classification method, and municipal solid waste can be classified into kitchen waste, recyclable waste, hazardous waste, and other waste. By doing so, these provinces can effectively alleviate the pressure of municipal solid waste, increase the efficiency of recycling and reuse, and make municipal solid waste become a renewable resource. In our global model, the proportion of the increasing value of the TIP was insignificant. This may have resulted from regional heterogeneity, which was further analyzed in the clustering step. 

The age structure (AGE) and family size (FS) are important control variables and they showed significant results in the global model. In this paper, we used the population dependency ratio to represent the age structure of each province. The higher the population dependency ratio means the province has a larger proportion of elderly and children in its population. The age structure had a significant and negative influence on MSW generation. This may be because the population aged 15 to 59 years are the main force of household income and consumption, thus they will contribute a lot to MSW generation. Ghinea et al. [7] conclude that the population aged 15 to 59 years strongly and positively influence waste generation. This conclusion is the same as the negative correlation between the age structure and MSW in our study. Furthermore, the family size was positively and significantly correlated with MSW generation. Some random sampling field researches also support this result. Han et al. [5] and Trang et al. [16] find that the family size is positively correlated with the total amount and components of MSW, and they think both the family size and family structure can influence MSW generation. For example, a family with only the frugal elderly will generate less waste than a nuclear family. 

### 5.2. MSW Generation in Local Models

After clustering provinces based on the economic and consumption indicators, some interesting differences to the typical east–medium–west division were observed.

Some provinces like Qinghai and Sinkiang were grouped into the middle economic and consumption level, and Inner Mongolia was grouped into the high economic and consumption level. This is different from our previous understanding of the economic development level in China. This is because, although the total amount of GDP and consumption expenditure is lower, the population density is lower. Thus, these provinces with large areas and sparse populations were clustered into the more developed groups. In the three local models, PSE, per capita GDP, PD, and FC had a significant and positive effect on MSW generation, which could be explained as per the global model. 

Household expenditure on housing (HC) was significant in all three local models, but had different effects. In model 1 and model 3, HC’s coefficients were −2.364 and −0.434, respectively, indicating that HC had a negative impact on MSW generation in provinces at the high and low economic and consumption levels. As in the general model, HC might reduce the actual ability to purchase and consume goods, thereby reducing MSW generation. However, in local model 2, the coefficient of HC was 1.131, indicating HC had a positive impact on MSW generation in provinces at the middle economic and consumption level. This may be because the general level of HC in those provinces is much lower, compared to provinces at the high economic development level. For example, the house rental accounts for a lower proportion of total expenditure in provinces at the medium level. If residents’ housing expenditure is high, it often means that they have stronger economic ability to enjoy a higher standard of living. Therefore, in provinces where residential housing pressure is not very high, residents should be guided to consume rationally, save resources, and reuse the household waste. 

The TIP was another interesting factor. It showed an insignificant impact in the global model due to the regional heterogeneity. However, in local models its impact changed. From Table 6 we could see that TIP became significant in local model 1 and local model 3. In local model 3, TIP appeared to have a positive effect on MSW generation, which is similar to the conclusions of Wang and Geng [11]. As the goods provided by the tertiary industry are closely related to household income and expenditure, higher TIP means more goods have been consumed, leading to an increase in MSW generation. However, in local model 1, TIP’s coefficient was −7.353, indicating that TIP in provinces with high economic development and consumption levels was negatively correlated with MSW generation. TIP showed the opposite impact in high-level regions to that in low-level regions. This is due to the mature and sustainable waste collection system in the tertiary industry in high-level regions, as well as greater long-term emphasis and guidance on resource conservation. For example, the Beijing government requires that the tertiary industry improve the efficiency and level of renewable resources recovery, promote the integrated development of garbage classification and renewable resources recovery systems, and expand the community renewable resources recovery function coverage. In that case, increasing TIP may reduce MSW generation in provinces at high-level economic development. This points to the need for the establishment of a sound recycling and classification system in tertiary industries. At the same time, policies should focus more on the sustainable management of MSW. In high-level economic development regions, the government can consider investing waste-to-energy infrastructures and technologies. Firstly, landfill volume of MSW can be reduced to save land resources; additionally, MSW can be utilized as a form of renewable energy, to reduce the dependence of urban development on traditional energy sources.

The family size (FS) had a positive and significant impact on MSW in local model 1. Apart from possible reasons mentioned before, unique socioeconomic factors in high-level regions can also explain this. Due to the heavy pressure of household expenditure on housing in high consuming and economic provinces, many young people in high consuming and economic provinces choose to share one house for the cheaper rent. This shared lifestyle is different from the regular family lifestyle and the consumption behavior of each family member is very independent. Thus, the scale effect of consumption does not exist. Different from the results of Thanh et al. [13], the increase of family size in high-level regions will generate more waste. In addition, the age structure (AGE) had a negative and significant impact on MSW in local model 2, which can be explained as per the global model.

### 5.3. Future MSW Generation Forecasting in Liaoning, Hubei, and Sichuan Provinces of China

From Figure 4 we could see that although Liaoning, Hubei, and Sichuan provinces had similar starting points, Hubei province would generate the highest MSW volume and Sichuan province would have the fastest growth rate in five years. This result deserves serious consideration. We often thought that the degree of economic development and consumption were positively related to MSW generation. However, in our study, Hubei and Sichuan province, which belonged to the medium and low economic and consumption groups, showed the largest potential of MSW generation. In fact, this result is supported by the comparison between the MSW generation trend of China and OECD European countries. By recalling the OECD statistics in Figure 1, China has the fastest growth of MSW among all the OECD European countries since 1996 because of its huge economic potential and consumption levels, while the developed countries in OECD show almost no change in MSW generation volume. This can help us to understand our forecasting results. Hubei and Sichuan province have always been seen to have potential for economic development in China. To sum up, the developing provinces have large economic development potential, and these provinces may generate more MSW along with fast economic growth rates. Additionally, due to imperfect waste infrastructure and recycling systems, the recycling rates of MSW in developing provinces are low. All these points towards developing provinces generating large MSW volumes in the near future. In contrast, the developed provinces have a stable economic development rate and high recycling efficiency and rates of MSW. From the perspective of waste clearance volumes, the developed provinces will generate less MSW than developing provinces in the near future. 

With greater economic development, MSW will also expand significantly if no related waste management measures are implemented. Based on this, some regions that have less MSW pressure at present also need extra attention. In provinces with obvious economic development potential, such as Hubei province and Sichuan province, an MSW management strategy should be considered in advance to mitigate the threat of waste. Experience from MSW management in economically developed provinces can provide a good reference point. For example, Beijing implemented the Beijing Municipal Household Waste Management Regulations, requiring that relevant departments should publicize household waste management, popularize relevant knowledge, and enhance the public’s awareness of waste reduction and classification. The government has also emphasized financial support and guarantee for centralized transfer and treatment of household garbage and has focused on the protection of the surrounding environment.

## 6. Conclusions

In addition to typical influencing factors, this paper focuses more on the impact of China’s unique consumption patterns on MSW generation. In many provinces with high-level economic development, housing rent is a large proportion of total household expenditure. This may affect the actual purchasing power of residents, which in turn leads to differences in MSW generation among different regions. Therefore, are there any particular influencing factors that affect China’s MSW generation? How do the factors influence MSW generation? How will the MSW volume develop in the future? Following the methodology described above, different regression models were used in order to reveal the relationship between MSW generation and its influencing factors, both at the national level and at the provincial level. Based on the panel data of 30 provinces in China, a global model was built at the national level. From the regression result of the global model, we found that financial expenditure and consumption patterns have a significant effect on MSW generation. Particularly, the household expenditure on food has a significant positive impact on MSW generation, while household expenditure on housing plays the opposite role.

Due to the high heterogeneity among Chinese provinces, three well differentiated clusters of provinces have been derived according to economic and consumption levels. Three local models are developed to describe the characteristics of MSW generation in the three clusters. Household expenditure on housing and the tertiary industry proportion show different influencing directions on MSW generation in the different clusters. 

Three local models are then applied to forecast MSW generation volumes by taking Liaoning, Hubei, and Sichuan provinces as examples. The three provinces are respectively selected from the three different clusters and have similar baseline MSW volumes. By combining the regression models and GM(1,1), we can obtain the predicted value of MSW generation volumes. In five years, with the development of society and economy in China, the MSW generation in the three clustering provinces will continue to increase. By 2021, the MSW volumes in Hubei and Sichuan province will reach more than 10,000 ten thousand tons. The forecasting results of MSW generation provide insights to relevant policy-makers and departments. The MSW generation volumes will be greater than the predicted values and will place great pressure on the urban environment in China if there is no consideration of sustainable waste management. This trend will be much more obvious in the provinces with large economic development potential, and these provinces should implement measures to cope with the challenges and opportunities of future MSW generation. Based on the experience of MSW management from high-level economic development regions, measures can include improving the waste collection system, improving the efficiency of MSW recycling, investing in waste-to-energy infrastructure, and enhancing the environmental awareness of residents. 

In the future, more detailed analysis on China’s MSW composition and the economic-environmental evaluation of various MSW treatment technologies will be conducted. This will allow for determination of economical and sustainable methods of MSW management in China.

## Figures and Tables

**Table 1:** Typical socioeconomic factors of municipal solid waste (MSW) generation.

Independent Variables	Data Collection		Methods Used		References
	Level ^a^	Type ^b^	Models ^c^	Methods ^d^	
GDP	C	P	MLR and L	RA	Lu et al. [2]
	P	P	MLR	GMM	Wang and Geng [11]
	C	TM	F	LR and ANN	Chhay et al. [20]
Income	CT	S	MLR	ANOVA and RCA	Gu et al. [10]
	UR	CS	MLR	RS and Q	Bosire et al. [4]
	CT	P	MLR	EKC	Chen [9]
	CT	S	MLR	Q and OLS	Trang et al. [16]
Family size	CT	S	MLR	ANOVA and RCA	Gu et al. [10]
	CT	S	—	RS	Getahun et al. [21]
	UR	S	PA	CA and PA	Xu et al. [22]
Education	UR	S	PA	CA and PA	Xu et al. [22]
	CT	S	—	ST and RS and CA	Khan et al. [23]
Consumption expenditure	UR	CS	MLR	RS and Q	Bosire et al. [4]
	P	S	LR	CA	Han et al. [5]
Population density	CT	CS	SEM and SL	SE	Hage et al. [12]
	CT	P	MLR	CA and OLS	Oribe-Garcia et al. [6]
	P	SP	MLR and GWR	OLS and SAR	Keser et al. [24]
Retail sales	S	P	MLR	CA and ST	Hockett et al. [25]
Unemployment rate	CT	CS	SEM and SL	SE	Hage et al. [12]
	CT	S	MLR	CA and RA	Prades et al. [18]
Urbanization rate	CT	S	MLR	ANOVA and RCA	Thanh et al. [13]
Industrial structure	P	P	MLR	CA and GMM	Wang and Geng [11]

^a^ Data level: CT—city level; UR—Urban residential level; C—country level; S—state level; P—province level; ^b^ Data type: S—survey data; CS—cross-sectional data; P—panel data; SP—spatial data; TM—time-series data; ^c^ Models: LR—linear regression model; MLR—multiple linear regression model; PA—path analysis model; SEM—spatial error model; SL—spatial lag model; L—logistics model; F—fuzzy model; GWR—Geographically-Weighted Regression model. ^d^ Methods: ANN- Artificial Neural Network; OLS- Ordinary Least Square; ANOVA- Analysis of Variance; RCA—rank correlation analysis; RS—random sampling; Q—questionnaires; CA—correlation analysis; PA—path analysis; SE—spatial econometric; EKC—environmental Kuznets curve; RA—regression analysis; ST—Score tests; GMM—generalized moment method; SAR—spatial-auto regression; GWR—geographically-weighted regression.

**Table 2:** Methods used in municipal solid waste (MSW) forecasting models.

Methods	Models ^b^	Period Forecasted	Factors Involved ^a^									Reference
			P	GDP	CE	U	PD	FS	E	A	I	
Regression	MLR	2013–2023	√							√		Ghinea et al. [7]
	MLR	2016–2030	√	√	√							Chhay et al. [19]
	MLR	10 years		√			√	√				Beigl et al. [29]
Time-series	L and EGC	2013–2023										Ghinea et al. [7]
	SARIMA	Monthly and daily data										Navarro-Esbrı et al. [31]
	SARIMA	Month-scale										Xu et al. [30]
	GM(1,1)	2016–2030	√	√	√							Chhay et al. [19]
Grey models	GM(1,n)	2013–2030			√	√	√	√	√			Intharathirat et al. [32]
	GM(1,1)	2010–2020										Xu et al. [30]
Scenario analysis	—	2016–2030	√	√	√							Chhay et al. [19]
	—										√	Vučijak et al. [33]
Artificial neural network	ANFIS and SVM	Long term										Abbasi and El Hanandeh [27]

^a^ Factors involved: P—population; CE—consumption expenditure; U—urbanization; PD—population density; FS—family size; EM—employment; A-age; I—income; ^b^ Models: MLR—multiple linear regression models; L—linear model; Q—quadratic model; EGC—exponential growth curve; SARIMA—seasonal autoregressive moving average; GM(1,1)-Grey Model(1,1); GM(1,n)-Grey Model(1,n); ANFIS-Adaptive Network-based Fuzzy Inference System; SVM- Support Vector Machine.

**Table 3:** Description of statistical characteristics of variables.

Variables	Abbreviation	Unit	Mean	Standard Deviation	Min	Max
Municipal solid waste generation	MSW	10 thousand tons	566.7	411.5	63.6	2391
Financial expenditure for general public services	PSE	Billion yuan	295.9	174.2	36.4	979.7
Household expenditure on food	FC	yuan	4020.1	986	2600.4	7989.8
Household expenditure on clothing	CC	yuan	1177.5	257	452.9	1893.7
Household expenditure on housing	HC	yuan	1086.8	260.3	641.9	1841.9
Per capita GDP	PGDP	yuan per person	35,168.6	19,526	7940.8	103,588.6
Population density	PD	Square kilometers per person	2777.8	1226	622	5967
Tertiary industry proportion	TIP	%	42	9	28.6	80.2
Age structure	AGE	%	35.5	6.5	19.3	55.1
Family size	FS	people per household	3.1	0.3	2.3	3.9

**Table 4:** Results of correlation analysis.

	MSW	PSE	FC	CC	HC	PGDP	PD	TIP	AGE	FS
**MSW**	1.00									
**PSE**	0.854 ***	1.00								
**FC**	0.426 ***	0.267 ***	1.00							
**CC**	0.171 **	0.141 *	0.316 ***	1.00						
**HC**	0.475 ***	0.256 ***	0.765 ***	0.425 ***	1.00					
**PGDP**	0.390 ***	0.259 ***	0.763 ***	0.617 ***	0.868 ***	1.00				
**PD**	−0.071	0.0112	−0.162 **	−0.132 *	−0.23 ***	−0.188 **	1.000			
**TIP**	0.176 **	0.011	0.703 **	0.431 ***	0.579 ***	0.677 ***	−0.187 **	1.00		
**AGE**	−0.292 ***	0.023	−0.368 ***	−0.490 ***	−0.602 ***	−0.625 ***	0.091	−0.398 ***	1.00	
**FS**	−0.300 ***	−0.142 *	−0.510 ***	−0.687 ***	−0.606 ***	−0.694 ***	0.244 ***	−0.433 ***	0.668 ***	1.00

Statistical significance is indicated by: * *p* < 0.05, ** *p* < 0.01, *** *p* < 0.001.

**Table 5:** Test results for the global model and local models.

	Global Model	Local Model 1	Local Model 2	Local Model 3
Hausman test	χ^2^(7) = 110.33 ***	χ^2^(5) = 22.03 ***	χ^2^(6) = 27.30 **	χ^2^(4) = 30.43 **
Heteroscedasticity	χ^2^(30) = 2388.61 ***	χ^2^(10) = 661.93 ***	χ^2^(12) = 4711.72 ***	χ^2^(8) = 63.34 ***
Serial correlation	F(1, 29) = 53.66 ***	F(1, 9) = 29.907 ***	F(1, 11) = 46.750 ***	F(1, 7) = 46.324 ***
Cross-sectional dependence	3.234 ***	1.201	4.123 ***	1.593
Robust F Statistics	F(9, 29) = 35,847.45 ***	F(6, 9) = 1230.12 ***	F(7, 11) = 246.31 ***	F(5, 7) = 113.16 ***

Statistical significance is indicated by: *** *p* < 0.01, ** *p* < 0.05.

**Table 6:** Statistic results of the global model and local models.

Independent Variables	Global Model	Local Model 1	Local Model 2	Local Model 3
PSE	0.738 ** (3.87)	1.421 ** (4.71)	0.473 * (2.65)	0.223 (1.55)
FC	0.194 ** (2.98)	0.444 *** (3.42)	0.526 *** (7.76)	0.604 *** (7.16)
CC	−0.564 *** (−15.03)	–	−2.393 *** (−7.23)	–
HC	−0.620 *** (−3.79)	−2.364 *** (−5.84)	1.131 * (3.01)	−0.434 *** (−2.44)
PGDP	0.008 *** (6.06)	0.010 *** (9.59)	–	–
PD	−0.027 * (−2.51)	–	−0.051 ** (−4.24)	0.013 (1.91)
TIP	−2.254 (−1.55)	−7.353 * (−2.90)	0.320 (0.23)	2.193 * (2.94)
AGE	−3.066 * (−2.56)	–	−5.526 *** (−3.17)	–
FS	123.279 *** (5.14)	212.72 ** (4.53)	–	–
Constant	519.923 *** (5.64)	586.004 * (2.68)	653.157 *** (6.21)	−161.416 (−1.61)
R^2^	0.5380	0.7040	0.3443	0.7642

Statistical significance is indicated by: *** *p* < 0.01, ** *p* < 0.05, * *p* < 0.1.

**Table 7:** The forecasting ability of local models.

	Liaoning		Hubei		Sichuan	
	Real MSW	Predicted MSW	Real MSW	Predicted MSW	Real MSW	Predicted MSW
2007	771.4	686.5	673.2	719.4	548.5	599.4
2008	796.7	701.2	680.8	751.9	551	627.7
2009	813.3	746.3	680.6	771.4	590.1	644.1
2010	837.3	763.2	711.1	778.1	656	659.7
2011	876	791.8	736.3	819.0	669	698.4
2012	929.9	856.1	716.6	850.2	702.8	732.7
2013	927.1	889.1	745.8	855.3	750.7	764.1
2014	917.1	782.6	739.3	862.8	780	779.1
2015	933.2	701.9	832.2	868.9	823.6	796.4
2016	933.05	852.5	880.1	970.9	886.7	839.9
MAPE	**0.1098**		**0.1068**		**0.0520**	
R^2^	**0.88**		**0.93**		**0.99**	

^a^ MAPE—Mean Absolute Percentage Error. (Units: ten thousand tons).

## Appendix A

**Table A1:** Results of the multicollinearity test in the global model.

Variable	VIF	1/VIF
**PSE**	1.28	0.783
**FC**	4.11	0.243
**CC**	2.54	0.393
**HC**	5.40	0.185
**PGDP**	7.53	0.133
**PD**	1.14	0.876
**TIP**	2.63	0.380
**AGE**	2.46	0.406
**FS**	3.25	0.307
**Mean VIF**	3.37	

VIF-Variance Inflation Factor.

**Table A2:** Results of the multicollinearity test in the local models.

Variable	Local Model 1	Local Model 2	Local Model 3
**PSE**	1.89	1.65	1.68
**FC**	6.32	1.73	2.27
**CC**	–	1.86	–
**HC**	6.66	2.19	1.44
**PGDP**	4.33	–	–
**PD**	–	1.91	1.21
**TIP**	2.88	1.48	1.41
**AGE**	–	1.53	–
**FS**	3.65	–	–
**Mean VIF**	4.29	1.76	1.60
